# Increased Expression of *TICRR* Predicts Poor Clinical Outcomes: A Potential Therapeutic Target for Papillary Renal Cell Carcinoma

**DOI:** 10.3389/fgene.2020.605378

**Published:** 2021-01-11

**Authors:** Shuang Xia, Yan Lin, Jiaqiong Lin, Xiaoyong Li, Xuexian Tan, Zena Huang

**Affiliations:** ^1^Department of Cardiology, Guangdong Cardiovascular Institute, Guangdong Provincial Key Laboratory of Coronary Heart Disease Prevention, Guangdong Provincial People’s Hospital, Guangdong Academy of Medical Sciences, Guangzhou, China; ^2^Department of Nephrology, The Third Affiliated Hospital of Guangzhou Medical University, Guangzhou, China; ^3^Department of Medical Genetics, School of Basic Medical Sciences, Southern Medical University, Guangzhou, China; ^4^Department of Surgery, The Third Affiliated Hospital of Guangzhou Medical University, Guangzhou, China; ^5^Department of Pathology, The Third Affiliated Hospital of Guangzhou Medical University, Guangzhou, China; ^6^Department of General Medicine, Guangdong Provincial Geriatrics Institute, Guangdong Provincial People’s Hospital, Guangdong Academy of Medical Sciences, Guangzhou, China

**Keywords:** *TICRR*, papillary renal cell carcinoma, clinical outcome, Treslin, immune infiltration

## Abstract

**Background:** Papillary renal cell carcinoma (PRCC), although the second-most common type of renal cell carcinoma, still lacks specific biomarkers for diagnosis, treatment, and prognosis. TopBP1-interacting checkpoint and replication regulator (*TICRR*) is a DNA replication initiation regulator upregulated in various cancers. We aimed to evaluate the role of *TICRR* in PRCC tumorigenesis and prognosis.

**Methods:** Based on the Kidney Renal Papillary cell carcinoma Project (KIRP) on The Cancer Genome Atlas (TCGA) database, we determined the expression of *TICRR* using the Wilcoxon rank sum test. The biological functions of *TICRR* were evaluated using the Metascape database and Gene Set Enrichment Analysis (GSEA). The association between *TICRR* and immune cell infiltration was investigated by single sample GSEA. Logistic analysis was applied to study the correlation between *TICRR* expression and clinicopathological characteristics. Finally, Cox regression analysis, Kaplan–Meier analysis, and nomograms were used to determine the predictive value of *TICRR* on clinical outcomes in PRCC patients.

**Results:** *TICRR* expression was significantly elevated in PRCC tumors (*P* < 0.001). Functional annotation indicated enrichment with negative regulation of cell division, cell cycle, and corresponding pathways in the high *TICRR* expression phenotype. High *TICRR* expression in PRCC was associated with female sex, younger age, and worse clinical stages. Cox regression analysis revealed that *TICRR* was a risk factor for overall survival [hazard ratio (HR): 2.80, *P* = 0.002], progression-free interval (HR: 2.86, *P* < 0.001), and disease-specific survival (HR: 7.03, *P* < 0.001), especially in patients with male sex, age below 60 years, clinical stages II–IV and clinical T stage T1–T2.

**Conclusion:** Increased *TICRR* expression in PRCC might play a role in tumorigenesis by regulating the cell cycle and has prognostic value for clinical outcomes.

## Introduction

Renal cell carcinoma (RCC) is a life-threatening cancer worldwide, ranking sixth among the most commonly diagnosed cancers in men and 10th in women (Siegel et al., 2018). Papillary RCC (PRCC) is the second most common type of RCC, accounting for nearly 18% of RCC (Srigley et al., 2013). In addition, it is the most common histological subtype in pediatric RCC and has been reported in 18% of dialysis patients (Morabito et al., 2010). However, diagnosis, treatment, and prognosis of PRCC are now mostly based on histological features, whose subtyping remains unsatisfactory (Fernandes and Lopes, 2015). Recent studies have introduced several novel biomarkers for RCC diagnosis and prognosis, such as urine aquaporin-1 and perilipin-2 (Farber et al., 2017; Cao et al., 2018; Song et al., 2019). However, these studies were mostly carried out in patients with rough RCC or clear cell RCC, lacking specific result for PRCC. An immunohistochemical marker α-methylacyl coenzyme A racemase was used for identifying PRCC (Alshenawy, 2015), while it was unrelated with PRCC prognosis. Several mutated genes were proved to be associated with PRCC diagnosis and treatment, including *MET*, *NF2*, *SETD2*, and Nrf2 pathway genes. Unfortunately, they were not sensitive enough, as they were only found in about 10 to 15% of PRCC (Akhtar et al., 2019). Therefore, it is urgent to search for a more convincing and suitable biomarker for PRCC.

TopBP1-interacting checkpoint and replication regulator (*TICRR*), also known as Treslin, is a critical DNA replication initiation regulator mediated by cyclin-dependent kinases and a DNA damage checkpoint (Boos et al., 2011). Biologically, *TICRR* regulates the cell cycle via determining S-phase progression from the expression level to epigenetic control (Charrasse et al., 2017; Maya-Mendoza et al., 2018) and thus promotes DNA replication. Overexpression of *TICRR* has been observed in several cancers, such as breast invasive carcinoma and liver hepatocellular carcinoma (Yu et al., 2019). It is associated with tumorigenesis, resistance to chemotherapy, and poor clinical outcomes (Yu et al., 2019). However, the potential role and underlying mechanism of *TICRR* in PRCC is not clear yet.

Using the RNA sequencing and clinical data of PRCC patients retrieved from The Cancer Genome Atlas (TCGA) database, we carried out a bioinformatics analysis to identify the significance of *TICRR* in PRCC tumorigenesis and prognosis. We observed an overexpression of *TICRR* in PRCC and investigated its potential role in PRCC tumorigenesis. Next, we performed a correlation analysis between *TICRR* and several clinicopathological characteristics. Finally, we identified the diagnostic and prognostic values of *TICRR*. This study provides novel insight into the underlying mechanisms of PRCC tumorigenesis and revealed *TICRR* as a potential diagnostic and prognostic biomarker in PRCC.

## Materials and Methods

### Data Processing and Ethics Statement

We downloaded high-throughput sequencing RNA data [fragments per kilobase per million (FPKM) format] and corresponding clinicopathological information from the Kidney Renal Papillary cell carcinoma Project (KIRP) on the TCGA database^1^. Excluding three patients with incomplete clinicopathological information, a total of 288 PRCC patients were enrolled. RNA sequencing data were transformed from FPKM format to transcripts per million reads for this study. As the TCGA database is open to the public under specific guidelines, it confirms that all written informed consents were obtained before data collection.

### Differentially Expressed Genes in Papillary Renal Cell Carcinoma Tumors

In total, 288 PRCC patients were separated into high- and low-*TICRR* expression groups according to *TICRR* median value. The R package “DESeq2”(Love et al., 2014) was used to identify differentially expressed genes (DEGs) between the two groups by a two-tailed hypothetical test based on the negative binomial generalized linear models, where the log-fold change larger than 1.5 and an adjusted *P*-value less than 0.05 were set as thresholds. The R packages “pheatmap” (Kolde, 2019) and “EnhancedVolcano” (Blighe, 2019) were applied to present results as heatmaps and volcano plots.

### Functional Annotation of *TICRR*-Associated Differentially Expressed Genes in Papillary Renal Cell Carcinoma Tumors

The identified DEGs were then processed for functional annotation on the Metascape database^2^ and online tool (Zhou et al., 2019). Minimum counts larger than 3, enrichment factors larger than 1.5, and a *P*-value less than 0.01 were set as analysis thresholds. Further, the R package “clusterProfiler” (Yu et al., 2012) was utilized for the Gene Set Enrichment Analysis (GSEA) (Subramanian et al., 2005) of the DEGs in the two groups. In GSEA, C2: curated gene sets from MSigDB collections were selected as reference gene sets. In total, 404 clusters were identified; clusters with a false discovery rate (FDR) less than 0.25 and *P*-value less than 0.05 were identified as significant. Protein–protein interaction (PPI) networks were investigated based on STRING database^3^ (Szklarczyk et al., 2019) and visualized using Cytoscape software (v3.7.1) (Shannon et al., 2003).

### Association of *TICRR* and Immune Cell Infiltration in Papillary Renal Cell Carcinoma Tumors

First, we used the single sample GSEA method from the R package “GSVA” (Hänzelmann et al., 2013) to present infiltration enrichment of 24 common immune cells, including dendritic cells (DCs), immature DCs (iDCs), activated DCs (aDCs), plasmacytoid DCs (pDCs), T cells, T helper (Th) cells, type 1 Th cells (Th1), type 2 Th cells (Th2), type 17 Th cells (Th17), regulatory T cells (Treg), T gamma delta (Tgd), T central memory (Tcm), T effector memory (Tem), T follicular helper (Tfh), CD8 + T cells, B cells, neutrophils, macrophages, cytotoxic cells, mast cells, eosinophils, natural killer (NK) cells, NK 56- cells, and NK 56 + cells. Next, the association between *TICRR* expression and immune cell infiltration was evaluated by Spearman’s analysis, and the infiltration levels of immune cells were compared for high- and low-*TICRR* expression groups by Wilcoxon rank sum test.

### Correlation Analyses for *TICRR* Expression and Clinicopathological Characteristics of Papillary Renal Cell Carcinoma Patients

Clinicopathological characteristics were compared for high- and low-*TICRR* expression groups using the Wilcoxon rank sum test (continuous variables) or Pearson’s chi-square test (rank variables). The correlation between *TICRR* expression and clinicopathological characteristics was evaluated by logistic analysis.

### Clinical Significance of *TICRR* Expression in Papillary Renal Cell Carcinoma

TICRR expression was compared between PRCC tumors and pericarcinous tissues by receiver operating characteristic (ROC) analysis to test the predictive value of *TICRR* for PRCC diagnosis. Information on PRCC patients’ clinical outcome was obtained from a published study (Liu et al., 2018), including overall survival, progression-free interval, and disease-specific survival. Kaplan–Meier (K-M) analysis, univariate, and multivariate Cox regression analysis were employed for prognosis analysis. The R package “randomForest” (Svetnik et al., 2003) was used for random forest regression. The R package “rms” (Harrell, 2020) was used to construct nomograms and calibration plots. The R package “forestplot” (Max Gordon, 2020) was applied for the clinicopathological subgroup study. The above statistical analyses were all carried out by R (v4.0.0), with *P*-values less than 0.05 considered significant.

## Results

### Expression Profiles of *TICRR* in Different Cancers and Related Differentially Expressed Genes in Papillary Renal Cell Carcinoma

**FIGURE 1:**
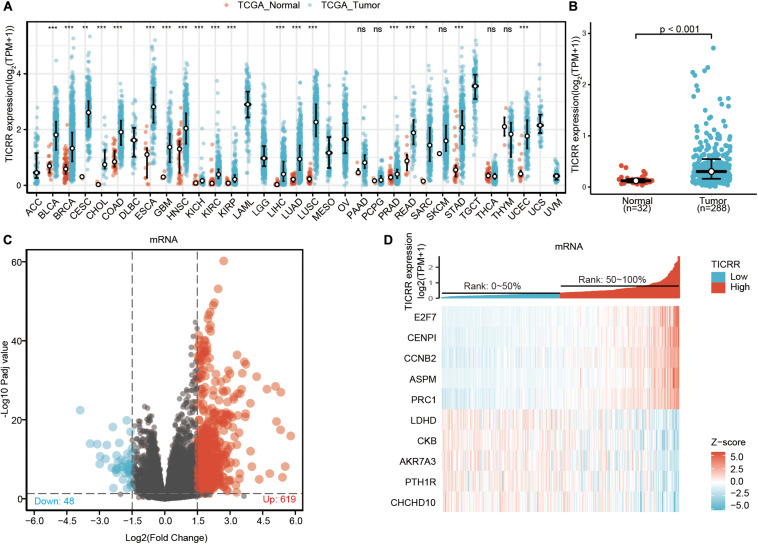
Differential mRNA expression profiles in papillary renal cell carcinoma (PRCC) patients stratified by *TICRR* levels. **(A)** The comparison of *TICRR* expression between tumor and pericarcinous tissue in different types of cancers based on TCGA database. ns, *P* ≥ 0.05; *P < 0.05; **P < 0.01; ***P < 0.001. **(B)** *TICRR* expression is higher in PRCC tumors than pericarcinous tissue. Based on the median *TICRR* level, 288 PRCC patients from The Cancer Genome Atlas –Kidney Renal Papillary cell carcinoma Project (TCGA-KIRP) were stratified into high- and low-*TICRR* expression groups. Shown are expression profiles of mRNA in two groups; and data are presented by volcano plots **(C)** and heatmaps **(D)**.

Based on TCGA database, we determined the expression of *TICRR* mRNA in different cancers. As shown in Figure 1A, among 33 cancer types, the *TICRR* was significantly highly expressed in 19 cancers, especially in tumors located in gastrointestinal and urogenital tracts. More specifically, *TICRR* expression was much higher in PRCC tumors than in pericarcinous tissues (*P* < 0.001, Figure 1B). Interestingly, in none of the investigated cancer profiles was *TICRR* expression significantly decreased.

Based on the median *TICRR* expression in PRCC tumors, 288 PRCC patients were stratified into two groups, high- and low-*TICRR* expression groups. We next compared mRNA, miRNA, and lncRNA expression between the two groups. Finally, 667 mRNAs (619 upregulated and 48 downregulated, Figure 1C), 2 miRNAs (2 upregulated, Supplementary Figure 1), and 341 lncRNAs (316 upregulated and 25 downregulated, Supplementary Figure 1) were recognized as DEGs (absolute value of fold change >1.5, *P* < 0.05) in the high-*TICRR* group. Representative DEGs were also illustrated by heatmaps (Figure 1D and Supplementary Figure 1).

### Functional Annotation of *TICRR*-Associated Differentially Expressed Genes in Papillary Renal Cell Carcinoma Tumors

**FIGURE 2:**
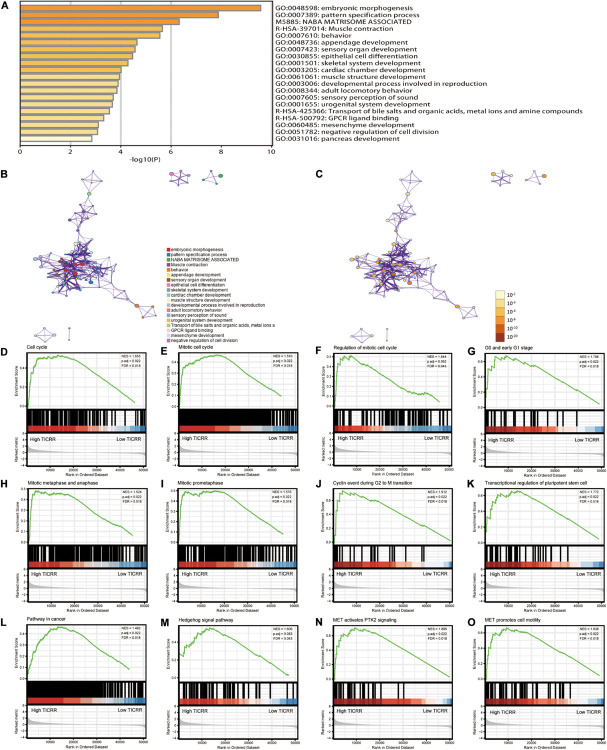
Functional annotation of differentially expressed genes (DEGs) in papillary renal cell carcinoma (PRCC) patients with distinct *TICRR* levels. According to the Metascape database, 691 differentially expressed mRNAs between high- and low-*TICRR* expression groups were used for functional annotation. All statistically enriched terms were identified and then hierarchically clustered into a tree **(A)** based on the threshold of kappa score as 0.3. Representative terms from the cluster were converted into a network layout **(B)**. The size of a node is proportional to the number of input genes that fall into that term, and the respective color represents its cluster identity. Terms with a similarity score >0.3 are linked by an edge (the thickness of the edge represents the similarity score). The same enrichment network presents nodes colored by the *P*-value **(C)**. **(D–O)** Representative Gene Set Enrichment Analysis of differentially expressed mRNAs between high- and low-*TICRR* expression groups.

In order to evaluate the function of *TICRR*-associated DEGs in PRCC patients, the software “Metascape” was applied. As presented in Figures 2A–C and Supplementary Table 1, we found that several PRCC-related pathways were enriched, including epithelial cell differentiation (GO: 0030855, *P* < 0.001, enrichment factor = 2.654, FDR = 0.037), urogenital system development (GO: 0001655, *P* < 0.001, enrichment factor = 3.448, FDR = 0.141), and negative regulation of cell division (GO: 0051782, *P* = 0.001, enrichment factor = 15.802, FDR = 0.266). Moreover, the GSEA showed *TICRR*-associated DEGs significantly enriched in cell proliferation related clusters (Figures 2D–K), such as mitotic cell cycle [normalized enrichment score (NES) = 1.510, adjusted *P* = 0.022, FDR = 0.018], cyclin events during G2 to M transition (NES = 1.912, adjusted *P* = 0.022, FDR = 0.018), mitotic metaphase and anaphase (NES = 1.524, adjusted *P* = 0.022, FDR = 0.018), and mitotic prometaphase (NES = 1.576, adjusted *P* = 0.022, FDR = 0.018). *TICRR*-associated DEGs were also enriched in cancer pathways (Figure 2L), especially the cell cycle-related Hedgehog signaling pathway (Figure 2M). More interestingly, *TICRR*-associated DEGs were associated with the activity of the MET gene (Figures 2N,O), which is usually involved in oncogenesis. We also constructed a PPI network for DEGs (Supplementary Figure 2), where the *TICRR* served as the hub gene related to another eight genes.

### Association of *TICRR* and Immune Cell Infiltration in Papillary Renal Cell Carcinoma Tumors

**FIGURE 3:**
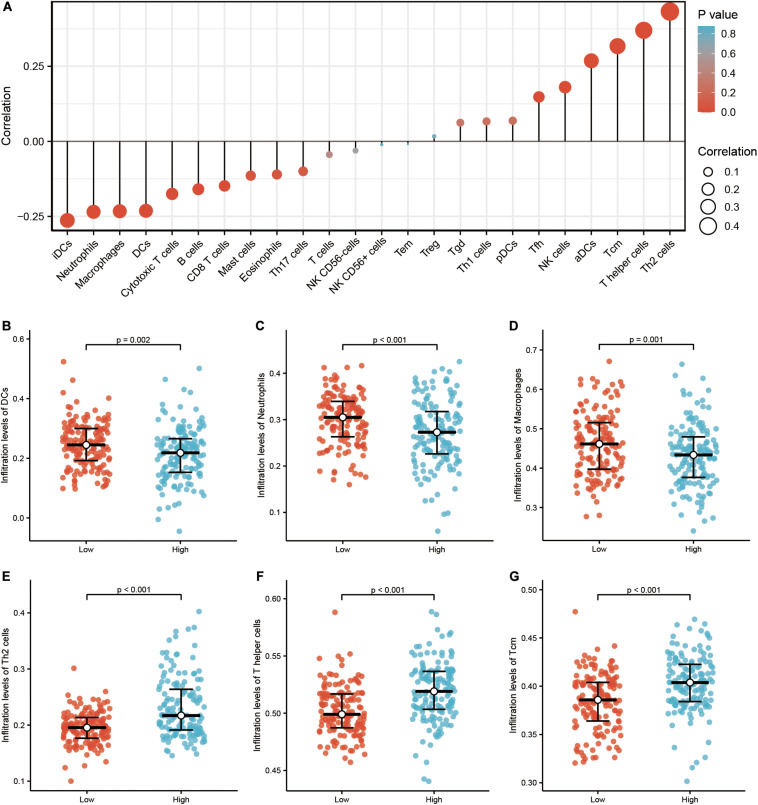
Correlation of immune cell infiltration and *TICRR* expression in papillary renal cell carcinoma (PRCC) patients. **(A)** Relationships among infiltration levels of 24 immune cell types and *TICRR* expression profiles by Spearman’s analysis. Shown is the comparison of infiltration levels of most correlated immune cells, including dendritic cells **(B)**, neutrophils **(C)**, macrophages **(D)**, type 2 T helper cells (Th2) cells **(E)**, Th cells **(F)**, and Tcm memory cells **(G)** between high- and low-*TICRR* expression groups. DCs, dendritic cells; aDCs, activated DCs; iDCs, immature DCs; pDCs, plasmacytoid DCs; Th, T helper cells; Th1, type 1 Th cells; Th2, type 2 Th cells; Th17, type 17 Th cells; Treg, regulatory T cells; Tgd, T gamma delta; Tcm, T central memory; Tem, T effector memory; Tfh, T follicular helper; NK, natural killer.

Infiltration of 24 immune cell types in PRCC was determined using the ssGSEA method first, and subsequently the association between *TICRR* and immune cell infiltration was investigated by Spearman’s analysis. As shown in Figure 3A, Tcm (*R* = 0.317, *P* < 0.001), Th cells (*R* = 0.317, *P* < 0.001), and NK cells (*R* = 0.180, *P* = 0.002) were all positively correlated with *TICRR* expression. However, DCs (*R* = −0.231, *P* < 0.001), macrophages (*R* = −0.233, *P* < 0.001), neutrophils (*R* = −0.235, *P* < 0.001), and B cells (*R* = −0.160, *P* = 0.007) showed a negative association with *TICRR*. More specifically, we evaluated the infiltration levels of six most relevant immune cells—DCs (Figure 3B), neutrophils (Figure 3C), macrophages (Figure 3D), Th2 cells (Figure 3E), Th cells (Figure 3F), and Tcm (Figure 3G)—in distinct *TICRR* groups, which showed results consistent with those in Figure 3A.

### Association of *TICRR* Expression and Clinicopathological Characteristics in Papillary Renal Cell Carcinoma Patients

We investigated the clinicopathological characteristics of PRCC patients with differential *TICRR* expression, as shown in Table 1. Compared with the low-*TICRR* group, patients in the high-*TICRR* group manifested a higher proportion of female sex, younger age, worse clinical stages, and more severe T and M stages. However, there was no significant difference in the distribution of clinical T stages, serum calcium concentration, hemoglobin level, or MET gene mutational status between two groups.

**TABLE 1:** Clinicopathological characteristics of PRCC patients with differential *TICRR* expression.

Characteristic	Level	Low-*TICRR* group (*n* = 144)	High-*TICRR* group (*n* = 144)
Sex (%)*	Female	24 (16.7%)	52 (36.1%)
Age (median [IQR])*		64.00 [57.00, 71.00]	59.00 [51.00, 69.00]
Race (%)	Asian	1 (0.7%)	5 (3.6%)
	Black or African American	30 (22.4%)	30 (21.9%)
	White	103 (76.9%)	102 (74.5%)
Smoker (%)		65 (52.0%)	65 (53.7%)
Clinical T stage (%)*	T1	80 (77.7%)	59 (60.2%)
	T2	13 (12.6%)	13 (13.3%)
	T3	10 (9.7%)	25 (25.5%)
	T4	0 (0.0%)	1 (1.0%)
Clinical N stage (%)*	N0	72 (93.5%)	60 (78.9%)
	N1	5 (6.5%)	14 (18.4%)
	N2	0 (0.0%)	2 (2.6%)
Clinical M stage (%)	M0	101 (96.2%)	98 (95.1%)
	M1	4 (3.8%)	5 (4.9%)
Clinical stage (%)*	Stage I	80 (78.4%)	58 (60.4%)
	Stage II	12 (11.8%)	9 (9.4%)
	Stage III	7 (6.9%)	22 (22.9%)
	Stage IV	3 (2.9%)	7 (7.3%)
Serum calcium (%)	Normal	69 (75.0%)	64 (72.7%)
	Elevated	2 (2.2%)	4 (4.5%)
	Low	21 (22.8%)	20 (22.7%)
Hemoglobin (%)	Normal	64 (61.0%)	48 (46.6%)
	Elevated	0 (0.0%)	1 (1.0%)
	Low	41 (39.0%)	54 (52.4%)
MET status (%)	Mut	6 (4.3%)	14 (10.1%)

IQR, interquartile range; PRCC, papillary renal cell carcinoma. *p < 0.05.

**FIGURE 4:**
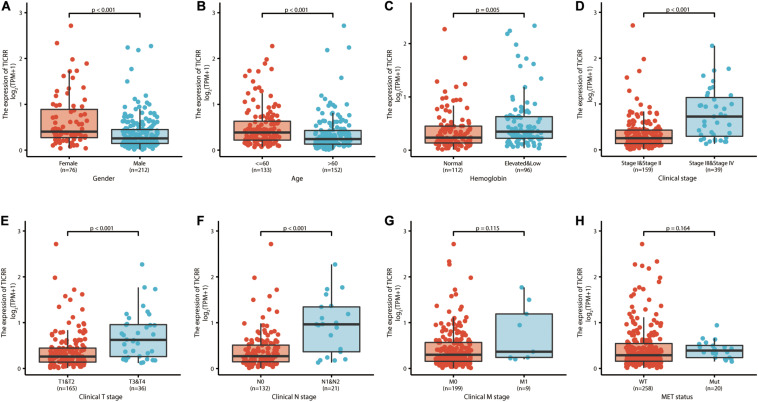
*TICRR* expression is associated with clinicopathological characteristics in papillary renal cell carcinoma (PRCC) patients. The Wilcoxon rank sum test was applied to analyze the association of *TICRR* expression with sex **(A)**, age **(B)**, hemoglobin level **(C)**, clinical stage **(D)**, clinical T stage **(E)**, clinical N stage **(F)**, clinical M stage **(G)**, and MET status **(H)**.

Further, we analyzed *TICRR* expression in patients with different clinicopathological characteristics. *TICRR* expression was significantly elevated in patients of female sex (Figure 4A), age below 60 years (Figure 4B), abnormal hemoglobin level (Figure 4C), clinical stages III and IV (Figure 4D), T stages T3 and T4 (Figure 4E), and N stages N1 and N2 (Figure 4F). As shown in Table 1, patients with different M stages (Figure 4G) and MET mutational status (Figure 4H) both shared similar *TICRR* expression levels. We also utilized logistics analysis to determine the correlation between *TICRR* expression and clinicopathological characteristics (Table 2). We found prominently positive correlations of *TICRR* expression with clinical stage (including T and N stages), hemoglobin, and female sex.

**TABLE 2:** Logistic regression analysis of association between clinicopathological characteristics and *TICRR* expression in PRCC patients.

Characteristic	Odds ratio (OR)	*P*-value
Clinical T stage (T3–T4 vs. T1–T2)	2.13 (1.29–3.79)	0.006
Clinical N stage (N1–N2 vs. N0)	2.64 (1.51–5.11)	0.002
Clinical M stage (M1 vs. M0)	1.60 (0.77–2.78)	0.115
Clinical stage (stage II–IV vs. stage I)	2.87 (1.61–5.72)	0.001
Serum calcium (abnormal vs. normal)	0.93 (0.48–1.59)	0.808
Hemoglobin (abnormal vs. normal)	1.74 (1.09–3.08)	0.032
MET status (Mut vs. WT)	0.71 (0.19–1.47)	0.501
Sex (Female vs. male)	2.22 (1.47–3.62)	<0.001
Age (>60 vs. ≤60)	0.66 (0.41–0.98)	0.054

*PRCC, papillary renal cell carcinoma; WT, wild type; Mut, mutation.*

### Predictive Value of *TICRR* for Papillary Renal Cell Carcinoma Diagnosis and Prognosis

**FIGURE 5:**
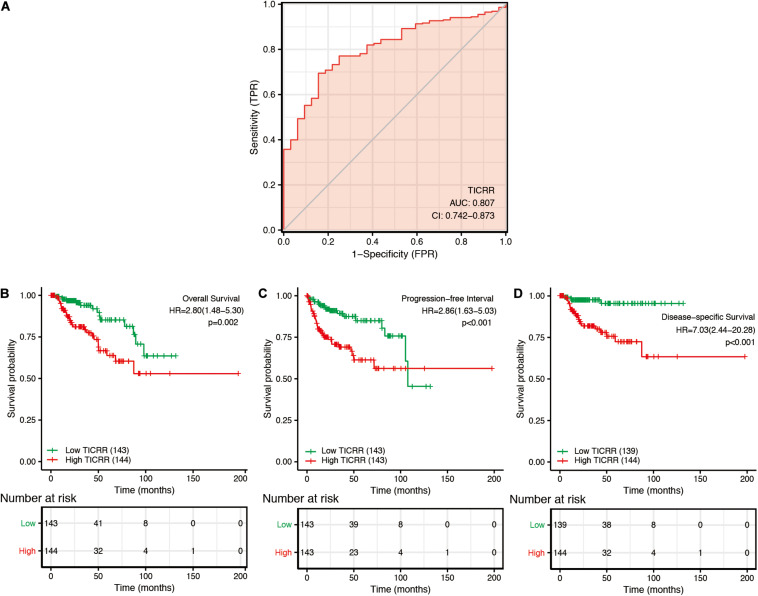
Predictive value of *TICRR* expression for diagnosis and clinical outcomes in papillary renal cell carcinoma (PRCC) patients. **(A)** Receiver operating characteristic (ROC) curve analysis evaluating the performance of *TICRR* for PRCC diagnosis. Shown are the Kaplan–Meier analyses comparing overall survival **(B)**, progression-free interval **(C)**, and disease-specific survival **(D)** between high- and low-*TICRR* expression groups.

In order to explore the clinical benefits of *TICRR* evaluation, we used a ROC curve to demonstrate its value on discriminating PRCC diagnosis. As the area under the curve (AUC) was 0.807, *TICRR* showed significant high sensitivity and specificity for PRCC diagnosis (Figure 5A). Next, K-M analyses were applied to verify the prediction of *TICRR* on clinical outcomes. As shown in Figures 5B–D, overall survival [hazard ratio (HR): 2.80, *P* = 0.002), progression-free interval (HR: 2.86, *P* < 0.001), and disease-specific survival (HR: 7.03, *P* < 0.001) for high-*TICRR* groups were all statistically worse than those for the low-*TICRR* group.

Moreover, we performed a multivariate Cox regression analysis to further evaluate the predictive value of *TICRR* on clinical outcomes. As shown in Table 3, *TICRR* expression was an independent risk factor for overall survival (HR: 3.862, *P* = 0.036) and disease-specific survival (HR: 4.705, *P* = 0.039) in multivariate Cox regression, although it did not provide any significant predictive ability for progression-free interval. Conversely, clinical stage, especially clinical N and M stages, also showed predictive advantages for clinical outcomes in multivariate Cox regression analyses. In order to evaluate the importance of each predictive factor for clinical outcomes, we carried out a random forest analysis to predict overall survival. The random forest model reached an overall percentage accuracy of 86.8%. As shown in Supplementary Figure 3, *TICRR* expression ranked second among the most important predictors of overall survival in PRCC patients.

**TABLE 3:** Cox regression analysis for clinical outcomes in PRCC patients.

Characteristics	HR for overall survival (95% CI)		HR for progression-free interval (95% CI)		HR for disease-specific survival (95% CI)	
	Univariate	Multivariate	Univariate	Multivariate	Univariate	Multivariate
Clinical T stage (T3–T4 vs. T1–T2)	4.687***	0.546	7.383***	1.923	8.926***	0.513
Clinical N stage (N1–N2 vs. N0)	10.637***	8.683*	17.022***	6.790**	19.162***	7.111*
Clinical M stage (M1 vs. M0)	38.111***	16.622**	10.324***	1.089	40.575***	20.996**
Clinical stage (stage II–IV vs. stage I)	5.123***	3.686	6.983***	1.976	27.918***	12.037*
Smoker (yes vs. no)	0.564		1.230		0.610	
Age (>60 vs. ≤60 years)	0.956		0.820		0.447*	1.321
Sex (male vs. female)	0.617		0.528		0.544	
Serum calcium (abnormal vs. normal)	1.659		1.180		1.749	
Hemoglobin (abnormal vs. normal)	4.381***	2.141	1.976*	2.137	3.174*	2.172
MET status (Mut vs. WT)	1.025		1.158		0.508	
Race (White vs. Black or African American and Asian)	0.921		0.863		0.891	
*TICRR* (high vs. low)	2.801**	3.862*	2.859***	2.496	7.029***	4.705*

HR, hazard ratio; PRCC, papillary renal cell carcinoma; WT, wild type; Mut, mutation; CI, confidence interval. *P < 0.05; **P < 0.01; ***P < 0.001.

**FIGURE 6:**
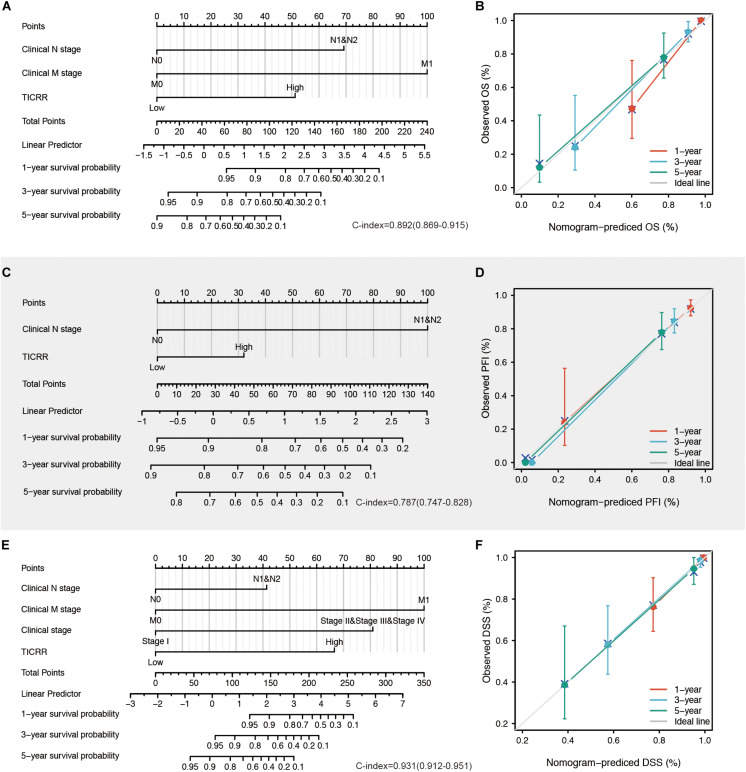
Construction and validation of nomograms based on *TICRR* expression. Shown are the nomograms constructed to establish *TICRR* expression-based risk scoring models for 1-, 3-, and 5-year overall survival **(A)**, progression-free interval **(C)**, and disease-specific survival **(E)**. Calibration plots validating the efficiency of nomograms for overall survival **(B)**, progression-free interval **(D)**, and disease-specific survival **(F)**. OS, overall survival; PFI, progression-free interval; DSS, disease-specific survival.

All the statistically significant prognostic factors in each multivariate Cox regression analysis were then used to construct a prognostic nomogram, and a calibration curve was drawn to test the efficiency of the nomogram. Clinical N and M stages, as well as *TICRR*, were included in the nomogram to predict overall survival, which had a C-index of 0.892 (Figure 6A). Clinical N and *TICRR* were included in a nomogram constructed to predict progression-free interval, which had a C-index of 0.787 (Figure 6C). Clinical stage, clinical N and M stages, and *TICRR* were used to construct a predictive nomogram for disease-specific survival, which had a C-index of 0.931 (Figure 6E). The calibration curves all presented desirable prediction of the three nomograms for the 1-, 3-, and 5-year clinical outcomes, with the exception of the 1-year prediction for overall survival, which was slightly underestimated (Figures 6B,D,F).

### Prognostic Performance of *TICRR* in the Papillary Renal Cell Carcinoma Clinicopathological Subgroups

**FIGURE 7:**
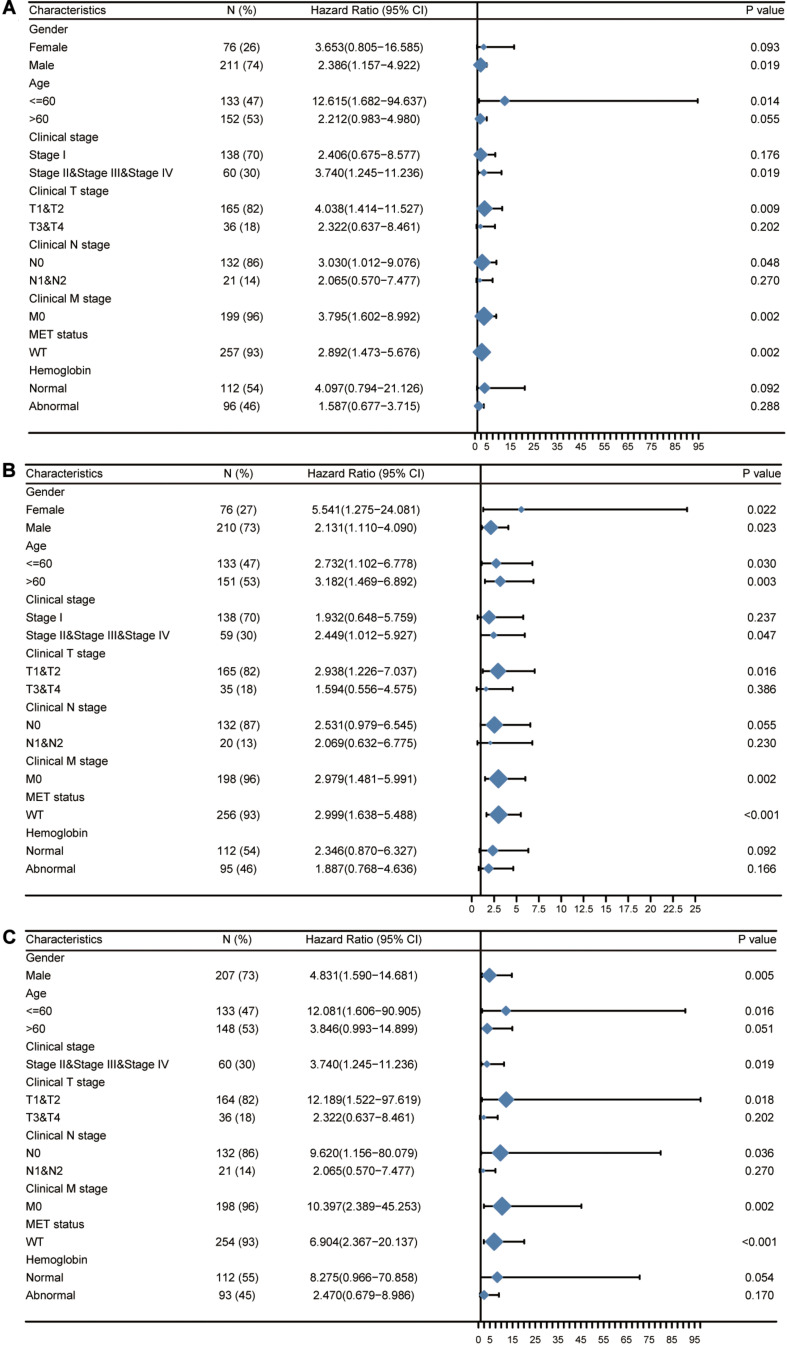
Prognostic performance of *TICRR* on clinical outcomes in different papillary renal cell carcinoma (PRCC) patient subgroups. Patients were divided into different subgroups according to sex, age, clinical stage, clinical TNM stage, MET status, and hemoglobin level. For each subgroup, the prognostic performance of *TICRR* on overall survival **(A)**, progression-free interval **(B)**, and disease-specific survival **(C)** were evaluated by Cox regression, and the results are presented as hazard ratio. The bar represents the 95% confidence interval of hazard ratio, the diamond’s size represents the significance of *TICRR*’s performance.

**FIGURE 8:**
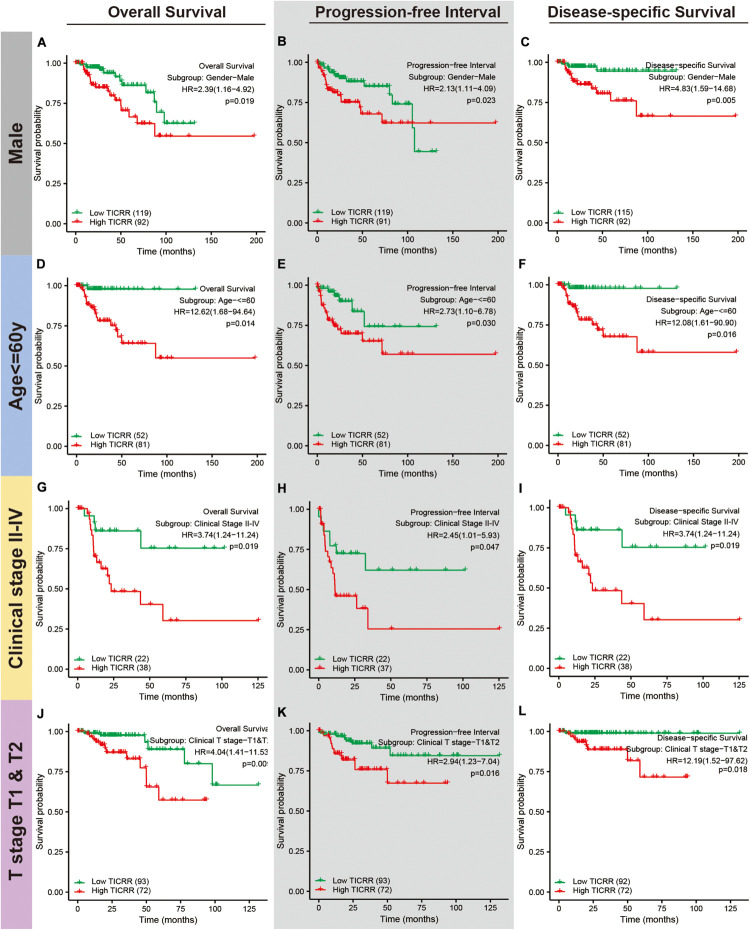
Distinct clinical outcomes based on *TICRR* expression in papillary renal cell carcinoma (PRCC) patient subgroups. Kaplan–Meier analysis showing the comparison of overall survival **(A,D,G,J)**, progression-free interval **(B,E,H,K)**, and disease-specific survival **(C,F,I,L)** between high- and low-*TICRR* expression groups in several PRCC patient subgroups, including male sex **(A–C)**, age below 60 years **(D–F)**, clinical stage II–IV **(G–I)**, and T stages T1–T2 **(J–L)**.

Next, we attempted to determine the predictive value of *TICRR* for clinical outcomes in several clinicopathological subgroups. We carried out Cox regression analyses in specific subgroups (Table 4). The results were also presented as forest plots (Figure 7). As shown in the forest plot in Figure 7A, *TICRR* was a significant risk factor for overall survival in patients of male sex (HR = 2.386, *P* = 0.019), age below 60 years (HR = 12.615, *P* = 0.014), clinical stage II–IV (HR = 3.740, *P* = 0.019), clinical T stages T1 and T2 (HR = 4.038, *P* = 0.009), clinical N0 stage (HR = 3.030, *P* = 0.048), clinical M0 stage (HR = 3.795, *P* = 0.002), and wild-type MET gene status (HR = 2.892, *P* = 0.002). Similar observations occurred for progression-free interval (Figure 7B) and disease-specific survival (Figure 7C). As there were few patients with clinical M1 stage (9 patients, occupying 4% of the sample) and MET mutation (20 patients, 7% of the sample), the subgroup analyses for clinical M1 stage and MET mutational status could not be performed. We also presented K-M analyses for clinical outcomes (overall survival, progression-free interval, and disease-specific survival) in the following four representative subgroups: male sex, age below 60 years, clinical stages II–IV, and T stages T1 and T2 (Figure 8). All the results demonstrated significantly better clinical outcomes in the low-*TICRR* expression groups.

**TABLE 4:** Prognostic performance of *TICRR* on clinical outcomes in PRCC patient subgroups by Cox regression analysis.

Characteristics	*N* (%)	HR for overall survival (95% CI)	HR for progression-free interval (95% CI)	HR for disease-specific survival (95% CI)
**Sex**				
Female	76 (26)	3.653 (0.805–16.585)	5.541 (1.275–24.081)*	N.A.
Male	211 (74)	2.386 (1.157–4.922)*	2.131 (1.110–4.090)*	4.831 (1.590–14.681)**
**Age**				
≤60	133 (47)	12.615 (1.682–94.637)*	2.732 (1.102–6.778)*	12.081 (1.606–90.905)*
>60	152 (53)	2.212 (0.983–4.980)	3.182 (1.469–6.892)**	3.846 (0.993–14.899)
**Clinical stage**				
Stage I	138 (70)	2.406 (0.675–8.577)	1.932 (0.648–5.759)	N.A.
Stage II–IV	60 (30)	3.740 (1.245–11.236)*	2.449 (1.012–5.927)*	3.740 (1.245–11.236)*
**Clinical T stage**				
T1–T2	165 (82)	4.038 (1.414–11.527)**	2.938 (1.226–7.037)*	12.189 (1.522–97.619)*
T3–T4	36 (18)	2.322 (0.637–8.461)	1.594 (0.556–4.575)	2.322 (0.637–8.461)
**Clinical N stage**				
N0	132 (86)	3.030 (1.012–9.076)*	2.531 (0.979–6.545)	9.620 (1.156–80.079)*
N1–N2	21 (14)	2.065 (0.570–7.477)	2.069 (0.632–6.775)	2.065 (0.570–7.477)
**Clinical M stage**				
M0	199 (96)	3.795 (1.602–8.992)**	2.979 (1.481–5.991)**	10.397 (2.389–45.253)**
M1	9 (4)	N.A.	N.A.	N.A.
**MET status**				
WT	257 (93)	2.892 (1.473–5.676)**	2.999 (1.638–5.488)***	6.904 (2.367–20.137)***
Mut	20 (7)	0.354 (0.022–5.659)	N.A.	N.A.
**Hemoglobin**				
Normal	112 (54)	4.097 (0.794–21.126)	2.346 (0.870–6.327)	8.275 (0.966–70.858)
Abnormal	96 (46)	1.587 (0.677–3.715)	1.887 (0.768–4.636)	2.470 (0.679–8.986)

HR, hazard ratio; CI, confidence interval; WT, wild type; Mut, mutation. *P < 0.05; **P < 0.01; ***P < 0.001.

## Discussion

In the present study, we focused on expression profiles, clinicopathological associations, and the clinical significance of a DNA replication initiation regulator, *TICRR*, in PRCC by analyzing datasets from the TCGA-KIRP. We observed prominent increased *TICRR* expression in PRCC tumors. DEGs related to higher *TICRR* levels were specifically enriched in cell cycle- and MET-associated pathways. We also revealed a marked association of *TICRR* expression with sex, age, and clinical stages in PRCC patients. Finally, we determined the predictive value of *TICRR* for overall survival, progression-free interval, and disease-specific survival in PRCC patients, especially in those of male sex, age below 60 years, and clinical stages II–IV and T stages T1–T2.

Uncontrollable DNA replication and thus cell proliferation are an essential mechanism in tumorigenesis. As a critical DNA replication regulator, *TICRR* plays an important role in several solid cancers (Yu et al., 2019). In our study, we found that *TICRR* was significantly elevated in several urogenital cancers, including PRCC, chromophobe renal carcinoma, renal clear cell carcinoma, bladder urothelial carcinoma, cervical squamous cell carcinoma, and in endocervical adenocarcinoma. Moreover, *TICRR* was also upregulated in tumors of other organs, such as breast invasive carcinoma, colon adenocarcinoma, and glioblastoma multiforme. Thus, *TICRR* may be a crucial hub gene in tumorigenesis.

Further, we attempted to describe the potential functions and mechanisms involving *TICRR* in PRCC. Based on results from previous studies (Bruck and Kaplan, 2015; Bruck et al., 2015), *TICRR* coordinates the assembly and activation of the eukaryotic replication fork helicase, which further unwinds double-stranded DNA and initiates DNA replication. In our study, based on functional annotation of *TICRR*-associated DEGs, epithelial cell differentiation and urogenital system development were closely associated with *TICRR* expression. Moreover, *TICRR* was associated with negative regulation of cell division. Based on additional GSEA, several cell cycle-related events were enriched in the high-*TICRR* group. The above data all provided evidence that *TICRR* functions as a critical DNA replication initiation regulator in PRCC. In a different study focusing on breast cancer, *TICRR* showed a similar effect on tumorigenesis, as silencing of *TICRR* significantly inhibited DNA replication, arrested cell cycle progression, and activated DNA damage (Yu et al., 2019). More interestingly, we found that patients in the high-*TICRR* group more frequently harbored MET mutations, which represents an appealing drug target given its prevalence in PRCC. The functional annotation analysis revealed that higher *TICRR* levels were associated with increased pathophysiological activity of the MET gene. Therefore, *TICRR* expression might be of great importance in PRCC tumorigenesis by affecting MET status and function.

We also revealed an underlying relationship between *TICRR* expression and immune cell infiltration. *TICRR* expression was negatively correlated with DCs, macrophages, and neutrophils. As the most effective antigen presenting cells, DCs activate CD 8 + T cells by cross-priming and further initiate anti-tumor immunity (Fu and Jiang, 2018). In the following immune response, neutrophils and macrophages work together against tumors (Qu et al., 2018). Moreover, neutrophils proved to be associated with better prognosis in different cancers (Donskov, 2013). Therefore, overexpressed *TICRR* seemed to dampen tumor immunity, help cancer cells escape from elimination, and finally accelerate tumorigenesis. On the other hand, we found a significantly positive correlation between *TICRR* expression and Tcm infiltration. Tumor-infiltrated Tcm cells have been reported in multiple cancers (Beckhove et al., 2004), which often exhibit dysfunctional phenotypes correlating with cancer progression (Reading et al., 2018). It can be explained that excessive neoantigen exposure caused functionally altered Tcm cells that skewed the anti-tumor response toward non-responsiveness (Merad et al., 2013).

Another issue of interest was the clinical significance of *TICRR* in PRCC. The ROC curve for *TICRR* discrimination of PRCC diagnosis had an AUC of 0.807, strongly suggesting that *TICRR* was a convincing biomarker for PRCC diagnosis. Moreover, we demonstrated that higher *TICRR* expression was correlated with several clinicopathological characteristics: female sex, younger age, abnormal hemoglobin, and worse clinical stages. As most of the above characteristics were risk factors for survival in PRCC patients (Fernandes and Lopes, 2015; Peng et al., 2018), we proposed TICRR as a marker for poor survival in PRCC. According to further Cox regression analyses and nomograms, *TICRR* presented satisfactory performance on clinical outcomes in PRCC. Patients with higher *TICRR* levels showed strikingly worse overall survival, progression-free interval, and disease-specific survival. This prognostic value of *TICRR* seemed to be more prominent in patients with specific features: male sex, age below 60 years, and clinical stages II–IV and T stages T1–T2. Using an online tool LOGpc (Long-term Outcome and Gene Expression Profiling Database of pan-cancers)^4^, *TICRR* was proved to be associated with lower overall survival in other tumors, such as renal clear cell carcinoma (Xie et al., 2019a), adrenocortical carcinoma (Xie et al., 2019b), breast invasive carcinoma (Yan et al., 2019), and lung cancer (Yan et al., 2020). The universal upregulation and predictive performance of *TICRR* indicated a possibility that it could represent a common prognostic biomarker for these cancer types.

Although we uncovered a potential mechanism for *TICRR* activity in PRCC tumorigenesis and its predictive value in PRCC clinical outcomes, our study still presented several limitations. First, because of the incomplete information about treatments and corresponding responses, we could not evaluate a specific role for *TICRR* in PRCC treatment. Second, we mainly focused on the RNA sequencing data from the TCGA database; thus, we were unable to provide information on relative protein levels or downstream pathways involving *TICRR*. Thus, these will remain areas for further *in vivo* and *in vitro* studies concentrating on the direct mechanism of *TICRR* activity in PRCC.

## Conclusion

Increased *TICRR* expression in PRCC might play a role in tumorigenesis by regulating cell cycle and exhibiting prognostic value for clinical outcomes. This study sheds light on *TICRR* as a potential therapeutic target for PRCC.

## Data Availability Statement

Publicly available datasets were analyzed in this study. This data can be found here: https://portal.gdc.cancer.gov/.

## Ethics Statement

All the data were collected and downloaded from TCGA database. As TCGA database is open to the public under specific guidelines, it is confirmed that all written informed consents were achieved. The patients/participants provided their written informed consent to participate in this study.

## Author Contributions

SX and YL: project investigation. XT: methodology. JL: writing–original draft. XL: writing–review and editing. ZH: project administration and supervision. All authors contributed to the article and approved the submitted version.

## Conflict of Interest

The authors declare that the research was conducted in the absence of any commercial or financial relationships that could be construed as a potential conflict of interest.

## Footnotes

1 https://portal.gdc.cancer.gov/

2 http://metascape.org

3 http://string-db.org

4 http://bioinfo.henu.edu.cn/DatabaseList.jsp

## Supplementary Material

Supplementary Figure 1 Differential non-coding RNA expression profiles in PRCC patients stratified by *TICRR* levels. Expression profiles of miRNAs **(A,C)** and lncRNAs **(B,D)** in two groups are presented by volcano plots **(A,B)** and heatmaps **(C,D)**.

Supplementary Figure 2 Protein-protein interaction networks of DEGs in PRCC patients with high- and low-*TICRR* expression levels. Based on the 691 differentially expressed mRNAs between high- and low- *TICRR* expression groups, we analyzed interactions using the STRING database, where the interaction threshold was set as 0.4. The line represents protein-protein interactions. The darker the filling color, the more mRNA interactions.

Supplementary Figure 3 Mean decrease Gini plot for important indexes associated with overall survival in PRCC patients. The random forest model was used to rank significant indexes, enrolling age, sex, smoking history, serum calcium level, hemoglobin level, TMN stage, clinical stage, and *TICRR* expression.

Supplementary Table 1 Top 20 clusters in pathway and process enrichment analysis of DEGs in PRCC patients with distinct *TICRR* levels.
