# Supplementary figures and images for: Increased Expression of TICRR Predicts Poor Clinical Outcomes: A Potential Therapeutic Target for Papillary Renal Cell Carcinoma
Source: Front Genet. 2021 Jan 11;11:605378. doi: 10.3389/fgene.2020.605378 (PMC7831611; doi:10.3389/fgene.2020.605378)

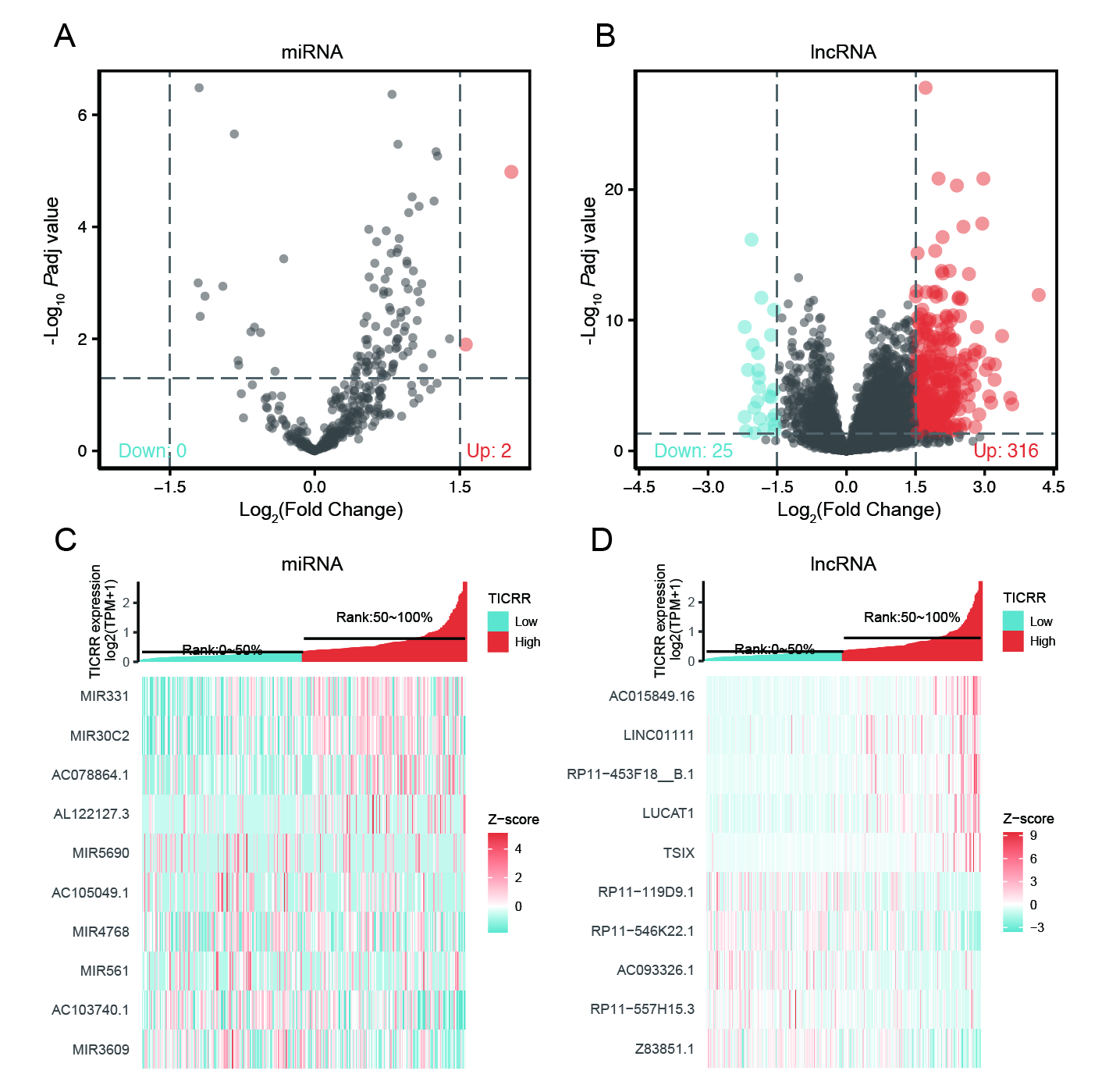

Supplement: Supplementary Figure 1 — Differential non-coding RNA expression profiles in PRCC patients stratified by TICRR levels. Expression profiles of miRNAs (A,C) and lncRNAs (B,D) in two groups are presented by volcano plots (A,B) and heatmaps (C,D). [file Image_1.TIF]

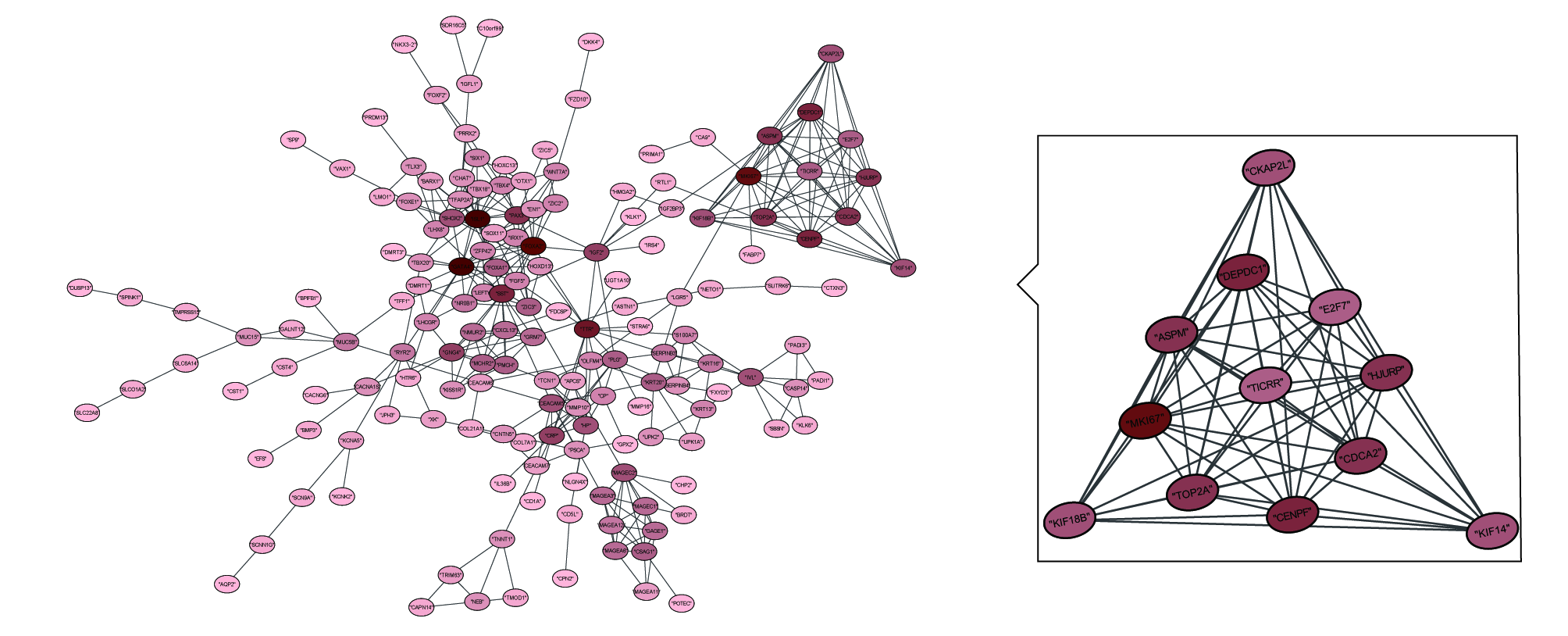

Supplement: Supplementary Figure 2 — Protein-protein interaction networks of DEGs in PRCC patients with high- and low-TICRR expression levels. Based on the 691 differentially expressed mRNAs between high- and low- TICRR expression groups, we analyzed interactions using the STRING database, where the interaction threshold was set as 0.4. The line represents protein-protein interactions. The darker the filling color, the more mRNA interactions. [file Image_2.TIF]

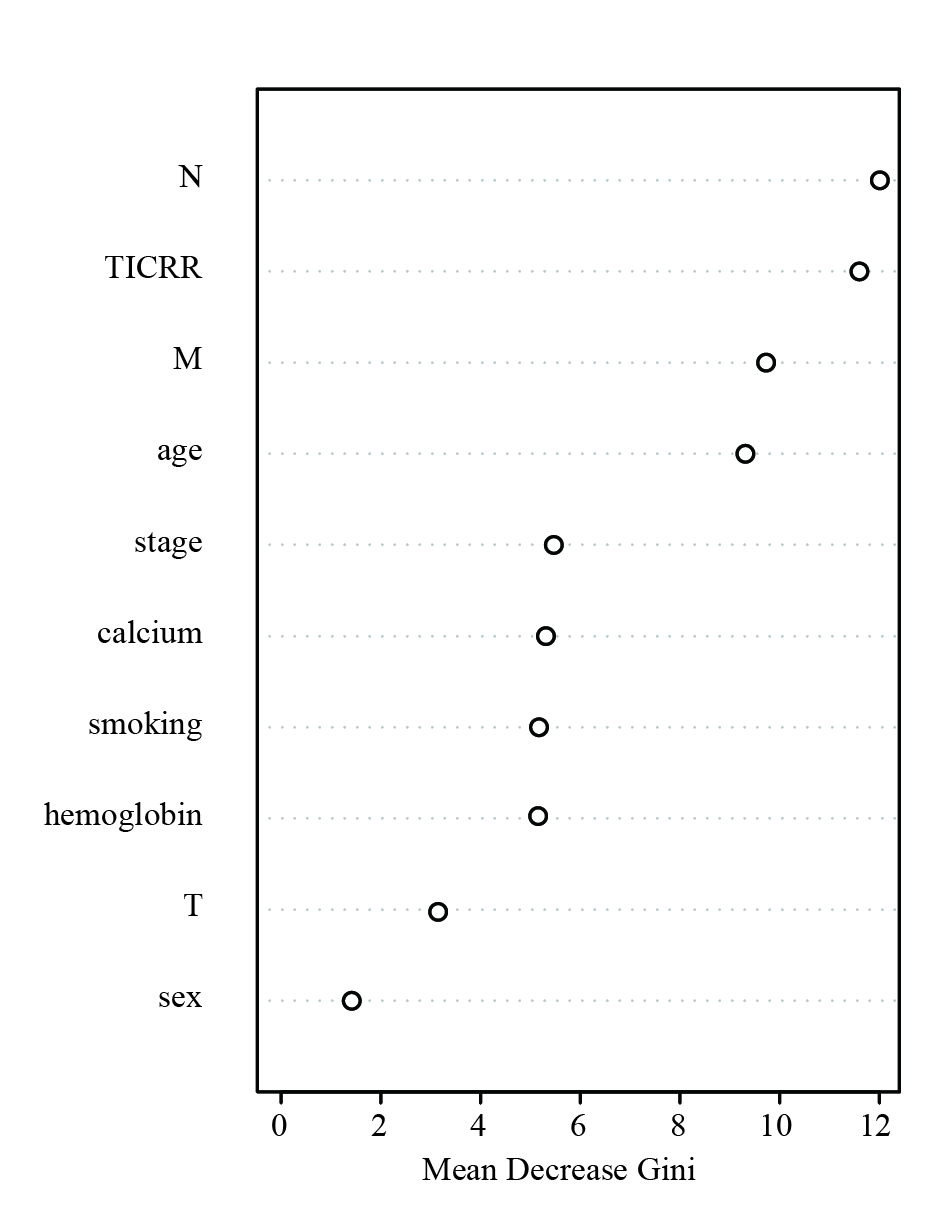

Supplement: Supplementary Figure 3 — Mean decrease Gini plot for important indexes associated with overall survival in PRCC patients. The random forest model was used to rank significant indexes, enrolling age, sex, smoking history, serum calcium level, hemoglobin level, TMN stage, clinical stage, and TICRR expression. [file Image_3.TIF]
