# Supplementary material for: Increased Expression of TICRR Predicts Poor Clinical Outcomes: A Potential Therapeutic Target for Papillary Renal Cell Carcinoma
Source: Front Genet. 2021 Jan 11;11:605378. doi: 10.3389/fgene.2020.605378 (PMC7831611; doi:10.3389/fgene.2020.605378)
Supplement: Supplementary Table 1 — Top 20 clusters in pathway and process enrichment analysis of DEGs in PRCC patients with distinct TICRR levels. [file Data_Sheet_1.PDF]

Table S1. Top 20 clusters in pathway and process enrichment analysis of DEGs in PRCC patients with distinct *TICRR* levels

Cluster	Description	P value	Enrichment Factor	FDR
GO:0048598	embryonic morphogenesis	<0.001	4.267	0.000
GO:0007389	pattern specification process	<0.001	4.464	0.000
M5885	NABA matrisome associated	<0.001	3.148	0.002
R-HSA-397014	muscle contraction	<0.001	5.496	0.005
GO:0007610	behavior	<0.001	3.227	0.006
GO:0048736	appendage development	<0.001	5.267	0.032
GO:0007423	sensory organ development	<0.001	3.080	0.032
GO:0030855	epithelial cell differentiation	<0.001	2.654	0.037
GO:0001501	skeletal system development	<0.001	3.047	0.054
GO:0003205	cardiac chamber development	<0.001	4.932	0.096
GO:0061061	muscle structure development	<0.001	2.665	0.106
GO:0003006	developmental process involved in reproduction	<0.001	2.638	0.116
GO:0008344	adult locomotory behavior	<0.001	7.585	0.124
GO:0007605	sensory perception of sound	<0.001	5.057	0.136
GO:0001655	urogenital system development	<0.001	3.448	0.141
R-HSA-425366	transport of bile salts and organic acids, metal ions and amine compounds	<0.001	6.693	0.155
R-HSA-500792	GPCR ligand binding	<0.001	2.842	0.194
GO:0060485	mesenchyme development	0.001	3.410	0.251
GO:0051782	negative regulation of cell division	0.001	15.802	0.266
GO:0031016	pancreas development	0.001	6.078	0.379

FDR: false discovery rate.
